# MiR-766-3p Suppresses Malignant Behaviors and Stimulates Apoptosis of Colon Cancer Cells via Targeting TGFBI

**DOI:** 10.1155/2022/7234704

**Published:** 2022-01-17

**Authors:** Jianchao Gao, Lexue Fei, Xiaotang Wu, Hua Li

**Affiliations:** ^1^Department of Gastrointestinal Surgery, TangShan Central Hospital, 063000 Tangshan, China; ^2^Department of General Surgery, The Second Hospital of Tangshan, 063000 Tangshan, China; ^3^Shanghai Engineering Research Center of Pharmaceutical Translation, 200231 Shanghai, China

## Abstract

**Background:**

MicroRNAs (miRNAs) can affect the progression of colon cancer cells. A variety of miRNAs, especially miR-766-3p, are proved to be abnormally expressed in colon cancer, but the molecular mechanism of miR-766-3p in this cancer has not yet been fully defined.

**Methods:**

Differentially expressed genes in the TCGA-COAD dataset were searched through bioinformatics analysis. MiR-766-3p and TGFBI mRNA levels were measured by qRT-PCR. TGFBI protein expression was measured via Western blot. Targeting relation between miR-766-3p and TGFBI was investigated by dual-luciferase reporter gene assay. Cell proliferation, invasion migration, and apoptosis were detected by cell functional assays.

**Results:**

MiR-766-3p was less expressed, while TGFBI was conspicuously highly expressed in colon cancer. MiR-766-3p high expression suppressed cell malignant behaviors and induced cell apoptosis in colon cancer. MiR-766-3p had a targeting relation with TGFBI verified by dual-luciferase assay. The cancer-suppressive impact of miR-766-3p overexpression was attenuated by overexpressing TGFBI.

**Conclusions:**

MiR-766-3p/TGFBI axis suppressed malignant behaviors and facilitated apoptosis of colon cancer cells. MiR-766-3p may be an underlying target for colon cancer.

## 1. Introduction

Colon cancer is the third most frequent cancer throughout the world, with more than 1,800,000 new cases and 881,000 deaths in 2018, accounting for approximately 1/10 of all cancer cases and deaths [1]. It has been found by epidemiological, clinical, pathological, and molecular genetic studies that most colon cancers originate from adenomas. One or more simultaneous adenomas are usually found in surgical specimens of colon cancer, and risk of colon cancer elevates pronouncedly with the increased number of adenomatous polyps [2]. Patients' cure rate is low since they are usually diagnosed at an advanced stage [3]. Although the treatment and diagnosis technologies of colon cancer are being constantly improved, nearly half of the patients have recurrence and metastasis [4]. Hence, to know the mechanism of colon cancer cells is vital for developing new diagnostic and therapeutic techniques.

MicroRNAs (miRNAs) can modulate expression of target genes by binding to the 3′-untranslated region (3′-UTR) of their mRNAs directly [5]. Abnormal miRNA expression may affect the pathogenesis of cancer [6–8]. The regulatory mechanisms of various miRNAs in colon cancer have been revealed in recent years. For instance, miR-378 is underexpressed in colon cancer, and miR-378 overexpression weakens proliferation and facilitates apoptosis of colon cancer cells [9]. MiR-137/TCF4 axis inhibits cell malignant behaviors in colon cancer [10]. The miR-144 restrains the growth of colon cancer by modulating SMAD4 [11]. Nevertheless, functions of miR-766-3p in colon cancer cells have not been studied yet.

TGFBI participates in various physiological processes, including differentiation, tumor progression, and metastasis [12]. Many reports have substantiated that TGFBI exerts a key role in cancer progression. TGFBI accelerates oral squamous cell carcinoma cell proliferation and is related to the dismal prognosis of patients [13]. TGFBI overexpression facilitates the proliferative and migrative abilities of glioma cells [14]. However, no consensus has been reached on whether and under what circumstances TGFBI functions as a pro-oncogenic or antioncogenic molecule. For instance, TGFBI can work as a tumor inhibitor or promoter in ovarian cancer [15]. TGFBI mRNA level is higher in colon cancer tissues than in noncancer tissues [16]. Secretion of TGFBI in colon cancer cells has association with cancer invasiveness and extravasation [17].

Herein, we detected the miR-766-3p level in colon cancer and explored the effects of miR-766-3p on tumor cell behaviors in colon cancer through functional experiments. Then, the downstream target of miR-766-3p was screened out. The present study confirmed that miR-766-3p targeted TGFBI to modulate malignant progression of colon cancer cells.

## 2. Materials and Methods

### 2.1. Bioinformatics Approaches

Expression profiles of mature miRNAs (normal, 8; tumor, 450) and mRNAs (normal, 41; tumor, 473) of colon cancer along with corresponding clinical information were obtained from TCGA (https://portal.gdc.cancer.gov/). Differential analysis was done by utilizing “EdgeR” package to acquire differentially expressed mRNAs (DEmRNAs) and miRNAs (DEmiRNAs) (thresholds: |logFC| > 2 and padj < 0.05). MiR-766-3p (the target miRNA) was identified by literature consultation. The target mRNAs of miR-766-3p were analyzed by mirDIP (http://ophid.utoronto.ca/mirDIP/index_confirm.jsp), miRTarBase (http://mirtarbase.mbc.nctu.edu.tw/php/index.php), and TargetScan (http://www.targetscan.org/vert_72/) databases. The predicted results were then overlapped with the DEmRNAs to obtain the target DEmRNAs. Finally, correlation analysis was utilized to identify the target mRNA.

### 2.2. Cell Culture

Normal colon epithelial cell line FHC (BNCC281458) and 4 colon cancer cell lines SW480 (BNCC337664), HCT116 (BNCC353646), Caco-2 (BNCC350772), and SW620 (BNCC100162) were from BeNa Culture Collection (BNCC, China). SW620, SW480, and Caco-2 were incubated in the L15 medium (Gibco; Thermo Fisher Scientific, Inc., USA), while HCT116 and FHC cells were placed in DMEM (Nacalai Tesque, Japan). Both the mediums contained 1% penicillin-streptomycin (Sigma, MO, USA) as well as 10% fetal bovine serum (FBS). The culture environment was 37°C and 5% CO_2_.

### 2.3. Cell Transfection

MiR-766-3p-mimic (miR-mimic) and control NC-mimic were from RioBio (Guangzhou, China). The pcDNA3.1-TGFBI vector (oe-TGFB1) was constructed by GenePharma (Shanghai, China), with empty vector pcDNA3.1 (oe-NC) as a negative control. Lipofectamine 2000 reagent (Invitrogen, Shanghai, China) was adopted for transfecting cells.

### 2.4. qRT-PCR

The mirVana miRNA Isolation Kit (Ambion, TX, USA) and TRIzol reagent (Invitrogen) were adopted for total RNA extraction. Then, reverse transcription of RNA was performed via the reverse transcription kit (Invitrogen) and miScript II RT kit. Real-time PCR was done using the SYBR® Premix Ex Taq™ II (Takara, Dalian, China) on the real-time PCR system (Bio-Rad Laboratories, CA, USA). The endogenous references were U6 and GAPDH.  Table 1 lists the used primers:

### 2.5. Western Blot

Isolation of total proteins was completed by the RIPA lysis method. After treated by SDS-PAGE, samples were transferred onto the PVDF membrane (MilliporeSigma). Then, after blocked with 5% skim milk, the membrane was cultivated with the primary antibodies: rabbit anti-TGFBI (1 : 2000, ab170874, Abcam, China) and rabbit anti-GAPDH (1 : 10,000, ab181602, Abcam, China). Afterwards, incubation of the membrane and corresponding secondary antibody, goat anti-rabbit IgG H and L (HRP) (ab6721, Abcam, China) was carried out. Finally, the Odyssey Infrared Imaging System (Li-CorBiosciences, NE, USA) was implemented to visualized the protein bands.

### 2.6. CCK-8 Assay

CCK-8 assay (Dojindo, Kumamoto, Japan) was utilized for cell proliferation detection. Caco-2 cells were cultured in a 96-well plate for 12 h. Corresponding oligonucleotides or plasmids were utilized to transfect cells. Then, CCK-8 solution was supplemented to the wells at the specified time points, followed by 1 h incubation at 37°C. Finally, the optical density (OD) value was detected at 450 nm.

### 2.7. Cell Invasion Assay

Transwell chamber (Millipore, USA) was implemented for cell invasion evaluation. First, the cells suspended in the L15 medium were added to the Matrigel-coated (BD, NJ, USA) upper compartment. The lower compartment was added with L15 containing 10% FBS. Then, cells failed to invade were removed after specified time. Meanwhile, the invading cells were handled with methanol for 10 min fixation and 0.1% crystal violet for 20 min staining. Cell count was performed under a light microscope.

### 2.8. Wound Healing Assay

Colon cancer cells were placed into a 12-well plate. After 24 h culture, the cell layer was scraped by a 10 *μ*L pipette tip to form a wound. The cells were rinsed and then incubated in a medium without serum. At specified time points, the photos of wound were taken with an optical microscope. At last, ImageJ software (National Institutes of Health, MD, USA) was adopted to analyze the results.

### 2.9. Cell Apoptosis Assay

Cells (1 × 10^5^ cells/100 *μ*L) were planted in a 6-well plate, treated twice with PBS 48 h following transfection, and then digested and resuspended by using 100 *μ*L of binding buffer. Density of cells was adjusted to 0.5 × 10^6^ cells/100 *μ*L. Next, Annexin V/FITC (5 *μ*L) was used to stain the cells in darkness for 10 min. Subsequently, 100 *μ*L of binding buffer was supplemented, and the plate was flicked to make the fluids mixed. Afterwards, cells staining was performed using 5 *μ*L PI for 5 min in darkness. The blank control group was set up, and the same experimental steps were performed without no staining. The apoptotic rate was detected within 1 h with FACScan flow cytometry (BD Biosciences).

### 2.10. Dual-Luciferase Reporter Gene Detection

The mutant-type (MUT) or wild-type (WT) TGFBI mRNA 3′-UTR sequence was inserted into downstream of psiCHECK vectors (Sangon Co., LTD, China). Then, miR-766-3p-mimic/NC-mimic and TGFBI-WT/TGFBI-MUT were transfected into colon cancer Caco-2 cells. Cells were harvested 48 h following transfection, and luciferase activity was analyzed by the Luc-Pair™ Duo-Luciferase Assay Kit (GeneCopoeia, China).

### 2.11. Statistical Analysis

Data processing was performed with GraphPad Prism 6.0 (La Jolla, CA). All data were presented as mean ± standard deviation. Data comparison was done by Student's *t*-test. *P* < 0.05 indicated a statistically remarkable difference.

## 3. Results

### 3.1. MiR-766-3p Is Lowly Expressed in Colon Cancer

**Figure 1:**
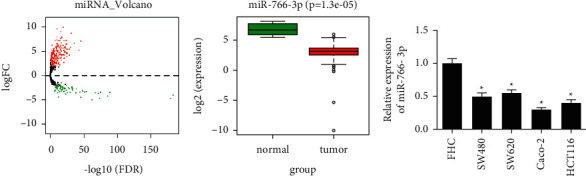
MiR-766-3p is less expressed in colon cancer cells. (a) Volcano map of DEmiRNAs in the TCGA database (red dots, prominently upregulated DEmiRNAs, and green dots, significantly downregulated DEmiRNAs). (b) Box plot of the miR-766-3p level in TCGA (green box, normal, and red box, tumor). (c) MiR-766-3p level in the normal colon epithelial cell line FHC and colon cancer cell lines SW480, SW620, Caco-2, and HCT116. *∗P* < 0.05.

Through differential analysis, 326 DEmiRNAs were obtained (Figure 1(a)). It is found through a literature review that miR-766-3p is less expressed and plays a pivotal part in various cancer tissues. Besides, expression analysis in TCGA indicated remarkable low level of miR-766-3p in colon cancer tissues (Figure 1(b)). Afterwards, result of qRT-PCR analysis uncovered that miR-766-3p was prominently less expressed in colon cancer cell lines (Figure 1(c)). It was suggested that miR-766-3p was downexpressed in colon cancer and may be a tumor inhibitor in cancer. Caco-2 with the lowest miR-766-3p level was utilized for the follow-up assays.

### 3.2. Forced Expression of MiR-766-3p Affects Cell Proliferation, Migration, Invasion, and Apoptosis in Colon Cancer

**Figure 2:**
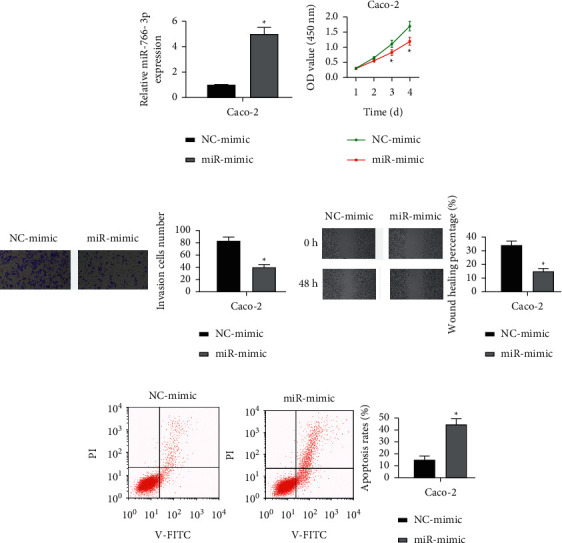
Forced expression of miR-766-3p affects cell malignant behaviors and apoptosis in colon cancer. (a) MiR-766-3p transfection efficiency in Caco-2 cells. (b) The proliferative ability of Caco-2 cells. (c) Invasive ability of Caco-2 cells (100×). (d) Migratory ability of Caco-2 cells (40×). (e) Apoptotic rate of Caco-2 cells. *∗P* < 0.05.

MiR-766-3p was overexpressed in Caco-2 cell line to study the effect of miR-766-3p on cell malignant behaviors in colon cancer. qRT-PCR result displayed that miR-766-3p expression was conspicuously high in the miR-mimic group (Figure 2(a)). CCK-8 result clarified that proliferation of colon cancer cells was weakened with overexpressed miR-766-3p (Figure 2(b)). The findings of transwell and wound healing assays denoted that miR-766-3p overexpression restrained cell invasion and migration in colon cancer (Figures 2(c) and 2(d)). Apoptosis assay exhibited that miR-766-3p overexpression dramatically stimulated apoptosis of colon cancer cells (Figure 2(e)). Thus, forced expression of miR-766-3p hampered cell malignant behaviors and stimulated cell apoptosis in colon cancer.

### 3.3. MiR-766-3p Targets and Inhibits TGFBI Expression in Colon Cancer

**Figure 3:**
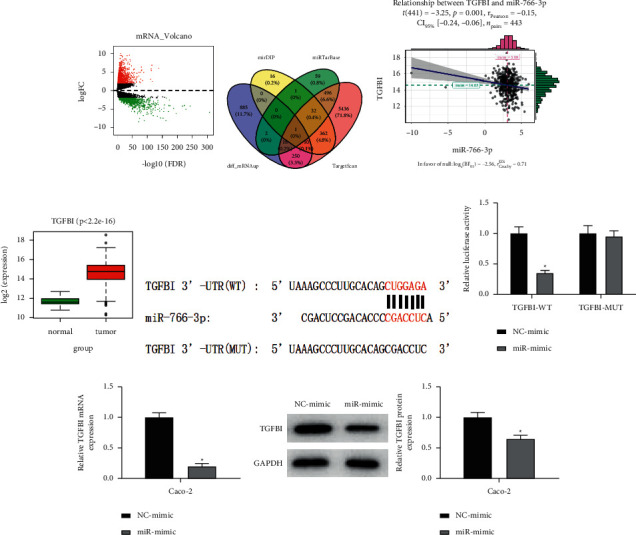
MiR-766-3p inhibits TGFBI expression in colon cancer. (a) Volcano map of DEmRNAs in normal and tumor groups. (b) Venn diagram of DEmRNAs and predicted target mRNAs. (c) Correlation of miR-766-3p and TGFBI. (d) Box plot of the TGFBI level in normal and tumor groups. (e) Binding of miR-766-3p on TGFBI 3′UTR predicted by bioinformatics analysis. (f) Dual-luciferase assay measuring the luciferase activity in different treatment groups of the cancer cell line Caco-2. (g) TGFBI mRNA level in colon cancer cells Caco-2 upon miR-766-3p overexpression. (h) TGFBI protein level in colon cancer cells upon miR-766-3p overexpression. *∗P* < 0.05.

2069 DEmRNAs were obtained in total through differential analysis (Figure 3(a)). The mRNAs targeted by miR-766-3p were analyzed by mirDIP, miRTarBase, and TargetScan databases and intersected with the 1166 upregulated DEmRNAs in the TCGA-COAD dataset. Finally, one target mRNA was obtained (Figure 3(b)). A negative correlation between miR-766-3p and TGFBI was revealed by correlation analysis (Figure 3(c)). Besides, TGFBI was notably highly expressed in colon cancer tissues in TCGA (Figure 3(d)). Afterwards, we predicted the binding sites between the two and discovered that they had a binding site (Figure 3(e)). Next, dual-luciferase assay result suggested that highly expressed miR-766-3p remarkably suppressed the luciferase activity of TGFBI-WT, whereas that of TGFBI-MUT did not change (Figure 3(f)). MiR-766-3p overexpression dramatically restrained the TGFBI mRNA level in colon cancer cells, as measured by qRT-PCR (Figure 3(g)). Western blot also exhibited that the TGFBI protein level was pronouncedly reduced by overexpressed miR-766-3p (Figure 3(h)). The above conclusions signified that miR-766-3p repressed TGFBI expression in colon cancer cells.

### 3.4. MiR-766-3p Hampers Cell Malignant Behaviors and Accelerates Cell Apoptosis in Colon Cancer by Modulating TGFBI

**Figure 4:**
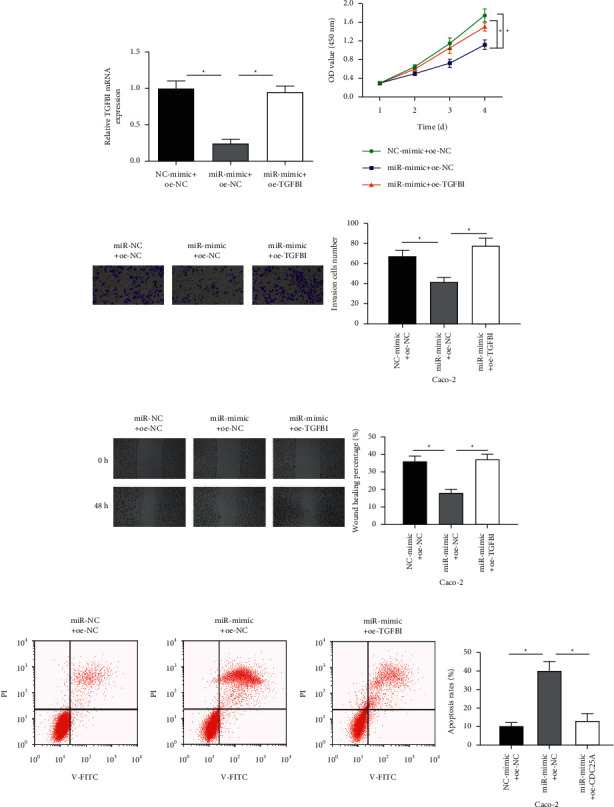
MiR-766-3p hampers cell malignant behaviors and stimulates cell apoptosis in colon cancer by downregulating TGFBI. (a) TGFBI mRNA expression in colon cancer cells Caco-2 (miR-NC + oe-NC, miR-mimic + oe-NC, and miR-mimic + oe-TGFBI). (b) Proliferation of Caco-2 cells. (c) Invasion of Caco-2 cells (100×). (d) Migration of Caco-2 cells (40×). (e) Apoptosis level of Caco-2 cells. *∗P* < 0.05.

To authenticate the impacts of miR-766-3p and TGFBI on colon cancer cells, we constructed a cell line with miR-766-3p overexpression (miR-mimic + oe-NC) and a cells line with simultaneous overexpression of TGFBI and miR-766-3p (miR-mimic + oe-TGFBI). According to the qRT-PCR result, TGFBI was dramatically lowly expressed in the miR-mimic + oe-NC group, while simultaneous overexpression of TGFBI and miR-766-3p recovered the TGFBI expression level (Figure 4(a)). It was illustrated that miR-766-3p had a suppressive effect on the expression of TGFBI. The result of CCK-8 demonstrated that when miR-766-3p was overexpressed, the proliferative ability of colon cancer cells was outstandingly reduced, while this inhibitory effect was attenuated by concurrently overexpressing TGFBI and miR-766-3p (Figure 4(b)). The results of cell invasion and wound healing assays indicated that miR-766-3p upregulation appreciably restrained cell migration and invasion of colon cancer cells, while such effect was weakened by simultaneously upregulating miR-766-3p and TGFBI (Figures 4(c) and 4(d)). Flow cytometry result clarified that overexpression of miR-766-3p markedly promoted cell apoptosis in colon cancer, while the upregulatory effect was suppressed by concurrently overexpressing miR-766-3p and TGFBI (Figure 4(e)). The above results proved that miR-766-3p hampered cell malignant phenotypes and accelerated cell apoptosis in colon cancer by downregulating TGFBI expression.

## 4. Discussion

The progression of colon cancer can be affected by oncogenes activation or the inactivation of antioncogenes [9]. The expression dysregulation of miRNA is closely linked with cancer progression [18]. Studies denoted that various miRNAs are differentially expressed in colon cancer. For instance, downexpressed miR-195 and miR-483 in colon cancer inhibit cancer cell proliferation and metastasis [19,20]. But how miR-766-3p functions in colon cancer remains to be investigated. Here, expression data of miRNAs in tumor tissues were downloaded from the TCGA-COAD dataset and analyzed through bioinformatics analysis, and miR-766-3p expression in tumor cells was tested via qRT-PCR. Based on the results, miR-766-3p was less expressed in colon cancer, and it was speculated that miR-766-3p might be an inhibitor in colon cancer.

The role of specific miRNAs in a certain cancer primarily depends on the gene expression in cancer [21]. Various assays have confirmed that the function of miR-766 differs in different cancer types. MiR-766 targets SOX6 to promote cell proliferation in colorectal cancer [22]. However, miR-766 can also induce p53 accumulation and G2/M arrest via modulating MDM4, which reduces cell proliferation in breast cancer [23]. MiR-766-3p is one of the miR-766 family members. You et al. [24] discovered that miR-766-3p/Wnt3a hampers liver cancer progression, but research of its specific role in other cancers is insufficient. A set of biological assays in this study authenticated that miR-766-3p upregulation inhibited cell malignant phenotypes and facilitated cell apoptosis in colon cancer, suggesting that miR-766-3p showed an inhibitory effect on colon cancer cells.

Through a conjoint analysis with related bioinformatics databases, we found that there was an interaction between miR-766-3p and TGFBI and that their expression was negatively correlated in colon cancer tissues. The dual-luciferase assay verified the targeting between miR-766-3p and TGFBI. TGFBI is also known as *β*ig-h3 or keratoepithelin [25]. TGFBI participates in the progression of tumors [26], and its expression is elevated in esophageal squamous cell carcinoma [27], gastric cancer [28], and bladder cancer [29]. However, reduced TGFBI expression has been observed in some malignancies, such as breast cancer and lung cancer [30, 31] compared with normal tissues. Thus, the function of TGFBI seems to greatly rely on the cellular background. In this study, TCGA analysis exhibited that TGFBI was dramatically overexpressed in colon cancer tissues. Consistent with our results, TGFBI plays a tumor-promoting role in CRC, and silencing TGFBI inhibits in vivo tumor growth and in vitro angiogenesis [32]. Studies have substantiated that TGFBI has a targeting relation with some miRNAs. For instance, Yan et al. [33] discovered that miR-21-5p/TGFBI induces nonsmall cell lung cancer cell proliferation. Bissey et al. [34] found a targeting association between miR-449b and TGFBI. Our experiments proposed that miR-766-3p and TGFBI had a targeting relation in colon cancer cells. The effect of simultaneous abnormal expression of miR-766-3p and TGFBI on the malignant phenotypes and apoptosis of colon cancer cells was studied to explore the regulatory relationship and the functional mechanism of miR-766-3p and TGFBI in colon cancer. The results certified that overexpression of TGFBI hindered the inhibitory impact of miR-766-3p forced expression on cell malignant behaviors and the promotive effect on cell apoptosis in colon cancer. The above results pointed out that miR-766-3p hampered cell malignant behaviors and promoted apoptosis in colon cancer through targeting TGFBI.

Viewed in total, miR-766-3p was less expressed in colon cancer cells. Functional assays in vitro denoted that miR-766-3p hindered cell malignant behaviors and stimulated cell apoptosis in colon cancer. The oncogene TGFBI was considered to target by miR-766-3p and was involved in colon cancer progression induced by miR-766-3p. These results concluded that miR-766-3p may be a new biomarker for colon cancer.

## Figures and Tables

**Table 1:** Information of primer sequences.

Gene	Primer sequences (5′⟶3′)
MiR-766-3p	F: 5′-ACTCCAGCCCCACAGC-3′
	R: 5′-GTCGTATCCAGTGCAGGGTCCGAGGTGCACTGGATACGACGCTGAGGC-3′

U6	F: 5′-GTGCTCGCTTCGGCAGC-3′
	R: 5′-GTCGTATCCAGTGCAGGGTCCGAGGTGCACTGGATACGACAAAATATGGAAC-3′

TGFBI	F: 5′-GTGCGGCTAAAGTCTCTCCA-3′
	R: 5′-AAGCCCTGGAAAACGCTGAT-3′

GAPDH	F: 5′-CGAGCCACATCGCTCAGACA-3′
	R: 5′-GTGGTGAAGACGCCAGTGGA-3′

## Data Availability

The data and materials used to support the findings of this study are available from the corresponding author upon request.

## Supplementary Materials

Supplementary Materials The protein bands of GAPDH and TGF*β*1.
