# Supplementary figures and images for: MiR-766-3p Suppresses Malignant Behaviors and Stimulates Apoptosis of Colon Cancer Cells via Targeting TGFBI
Source: Can J Gastroenterol Hepatol. 2022 Jan 17;2022:7234704. doi: 10.1155/2022/7234704 (PMC8786513; doi:10.1155/2022/7234704)

**GAPDH**


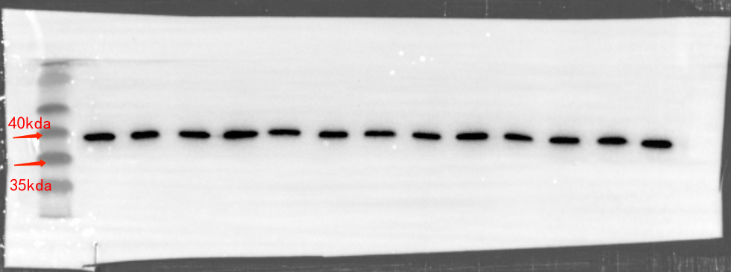


**TGFβ1**
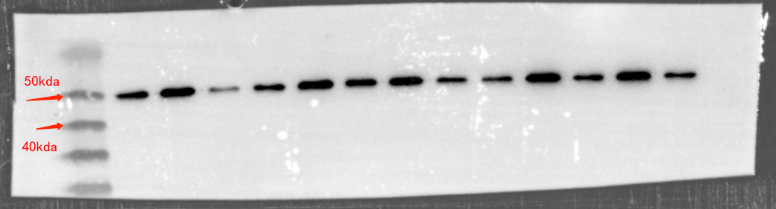

Supplement: Supplementary Materials — The protein bands of GAPDH and TGFβ1. [file 7234704.f1.docx]
